# Liposarcoma of the spermatic cord presenting as an inguinal hernia

**DOI:** 10.1016/j.ijscr.2020.09.195

**Published:** 2020-10-02

**Authors:** Eric Wetzel, Norair Adjamian, Graal Diaz, Shawn Steen, Janet Hobbs

**Affiliations:** a College of Osteopathic Medicine of the Pacific, Western University of Health Sciences, Pomona, CA, USA; b Graduate Medical Education, Community Memorial Health System, Ventura, CA, USA; c Department of General Surgery, Community Memorial Health System, Ventura, CA, USA

**Keywords:** Liposarcoma, Spermatic cord, Inguinal orchiectomy, Case report

## Abstract

**Introduction:**

Dedifferentiated liposarcoma is a rare, highly malignant, rapidly recurring tumor. When found in the spermatic cord, this tumor may deceptively present as an inguinal hernia, and due to its rarity, may not be readily identified.

**Presentation of case:**

We report a case of a 46-year old man with dedifferentiated liposarcoma presenting as a slow-growing right inguinal bulge. His surgical management included ipsilateral orchiectomy and excision with wide margins. He received adjuvant focused radiation therapy.

**Conclusion:**

Liposarcoma of the Spermatic Cord is a rare and highly malignant tumor that rapidly recurs. Therefore, a high degree of suspicion, regular surveillance and imaging are justified.

## Highlights

• Dedifferentiated liposarcoma is a highly malignant, rapidly recurring tumor.
• Dedifferentiated subtype is concerning due to aggressive behavior and rapid recurrence.
• Negative margins are an essential factor for recurrence-free survival.

## 1 Introduction

Liposarcoma of the spermatic cord is a rare diagnosis, which may present insidiously like an inguinal hernia. The incidence of malignant spermatic cord tumors is 0.3 cases per million, and of those, the most common type is liposarcoma [1]. Although they make up a minority of liposarcomas, dedifferentiated subtype is most concerning for aggressive behavior and rapid recurrence [1]. They present typically from the fifth to seventh decades of life, as either an inguinal hernia or paratesticular mass as they descend into the scrotum [[1], [2], [3]]. Given the rarity of this condition, most published data regarding management consists of case reports. From an academic medical system, we present a man in his late forties with an inguinal hernia and protracted two-year history of mild right lower quadrant pain. Consistent with published literature, his surgical management included radical orchiectomy with wide margins [[3], [4], [5]].

This work has been reported in line with the SCARE criteria [6]

## 2 Case report

**Image 1:**
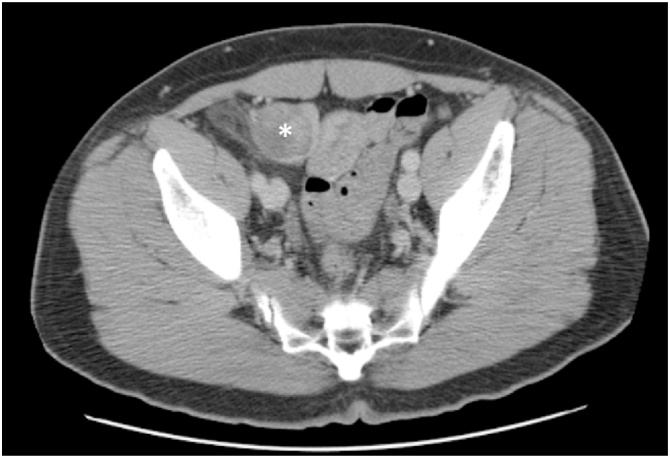
Asterisk represents Computed Tomography (CT) findings of a solid mass in the retroperitoneum, proximal to the inguinal canal.

**Image 2:**
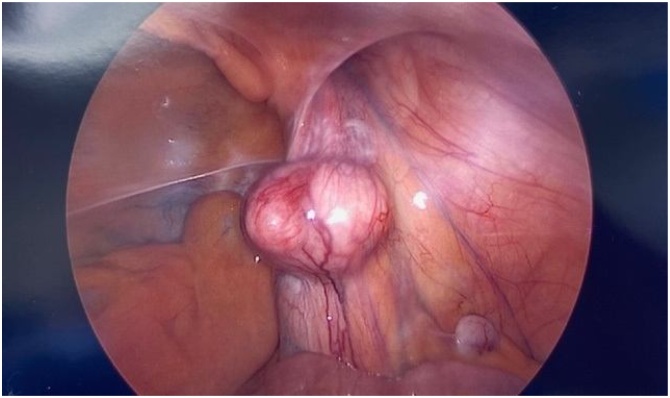
Laparoscopic visualization of firm mass coursing along the intra-abdominal.

**Image 3:**
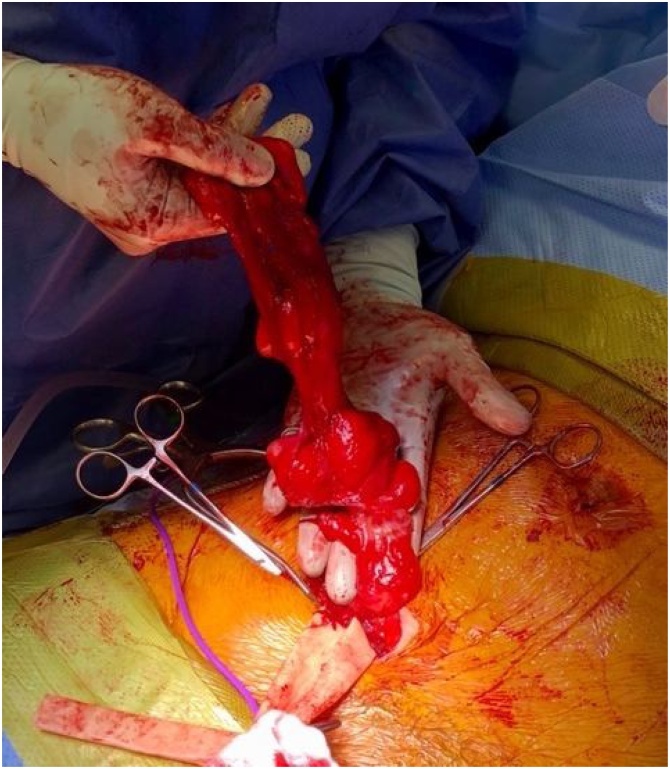
The initial excised 23 × 9 × 3 cm lobular adipose tissue, including the solid mass.

**Image 4:**
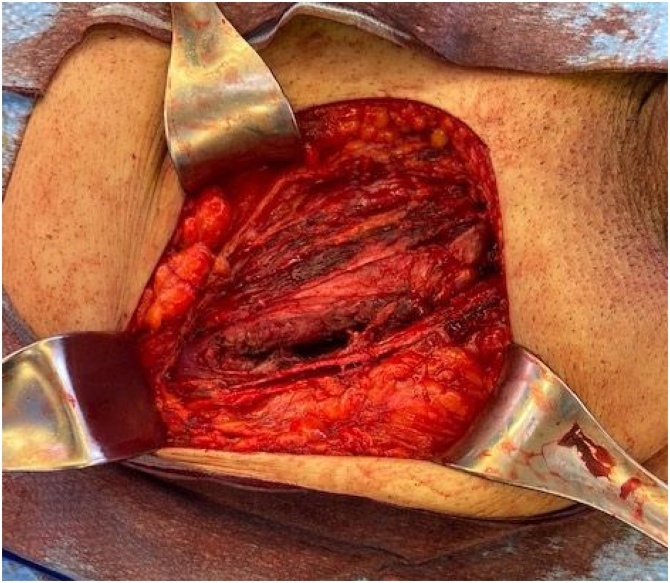
Resection of new abdominal wall margins and orchiectomy.

The patient is a 46-year-old male nonsmoker with no other medical problems. Two years prior to his current presentation, the patient was evaluated for right lower quadrant pain. Computed tomography (CT) image showed acute epiploic appendicitis of the cecum and a small fat-containing right inguinal hernia. No further treatment was pursued at that time. He had persistent symptoms, and two years later his primary care provider ordered an ultrasound, which showed a 3 × 3.4 × 3.6 cm complex heterogeneous mass superior to the bladder. Repeat CT scan confirmed a fat-containing right inguinal hernia and a solid mass just below the right inguinal canal, concerning for liposarcoma versus inflammatory process (Image 1). The patient underwent mass excision; given prominence of the mass in the retroperitoneum on imaging, the surgeon used a laparoscopic approach and visualized a 4 × 3 cm retroperitoneal mass tracking along the gonadal vessels (Image 2). The patient developed severe bradycardia during laparoscopy, and surgery was converted to an open approach. The area of the mass was accessed via a traditional right inguinal hernia incision and by enlarging the internal inguinal ring. The mass lesion was felt to be associated with the intraabdominal portion of the spermatic cord structures and also incorporated a large multilobular cord lipoma. The mass, along with the cord lipoma, was completely excised (Image 3). Surgical clips were placed along the resection margin of the retroperitoneal dissection. Intraoperative pathology could not confirm a malignant process so no further resection was undertaken at that time, and a standard Lichtenstein hernia repair was then completed, using polyprolene mesh. After outside consultation, final pathology results revealed an abnormal lobular adipose tissue specimen, measuring 23 × 9 × 3 cm. Within the larger fatty tumor was a well circumscribed firm 4 × 4 × 2.8 cm mass, which contained a high-grade spindle cell malignancy with pleomorphic cells and a high mitotic rate, consistent with dedifferentiated liposarcoma. MDM2 and CDK4 were positive in the well differentiated portions. Complete staging scans subsequently showed no evidence of metastatic disease to the intraabdominal organs or lungs. The deep retroperitoneal margin was positive for tumor cells, and the dedifferentiated portion was closely associated with the intraabdominal spermatic cord, necessitating wider resection margins and right orchiectomy. Three weeks from his index surgery the patient underwent repeat excision of two centimeter circumferential margins on the abdominal wall surrounding the internal inguinal ring, removal of additional retroperitoneal tissue overlying external iliac vessels, and finally a right radical orchiectomy (Image 4). The inguinal floor defect was repaired with biologic mesh. Pathology from the second operation revealed a microscopic focus of residual liposarcoma from the excised external iliac tissue, measuring 0.6 mm, 0.5 mm away from the cauterized edge. The remaining orchiectomy specimen and abdominal wall tissue were negative for malignancy. Once recovered from surgery, the patient underwent adjuvant radiation therapy of 25 fractions delivered at 50 Grays (Gy) in addition to a sequential boost of five fractions at 10 Gy. Six month follow-up magnetic resonance imaging (MRI) showed him to be free from disease recurrence. At that time, the patient remained active with biking and kayaking, denying any symptoms suggestive of hernia recurrence.

## 3 Discussion

Liposarcoma of the spermatic cord is a rare cause of inguinal hernia (0.1%), and most tumors of less than ten centimeters are found incidentally and not diagnosed preoperatively [7]. In general, dedifferentiated liposarcomas develop de novo and present as foci accounting for greater than twenty-five percent of a larger well differentiated mass. Although they make up a minority of cases, dedifferentiated lesions can develop in the setting of well differentiated tumors, accounting for 22 of the 155 cases analyzed by Henricks et al., with a mean interval of 7.7 years [7]. In our case, preoperative imaging studies showed a dense 3 × 3.8 × 3.1 cm mass and characteristics suspicious for liposarcoma. Of note, this patient had prolonged right lower quadrant pain and a CT scan showing possible early signs of tumor two years prior. These tumors can be slow-growing over 3–5 years and may appear as a “cord lipoma” on earlier CT scans [2,5]. Despite preoperative suspicion for malignancy, intraoperative pathology in our case was inconclusive, and margins were considered close enough to warrant a second surgery, which included consenting the patient for planned orchiectomy. Although only a small number of spermatic cord tumor cases have been published, all cases report surgical treatment with radical orchiectomy with high ligation of the spermatic cord and resection of involved abdominal wall. Negative margins are an essential factor for recurrence-free survival; following definitive histology, our patient returned to the operating room for resection with wider margins and ipsilateral orchiectomy. Findings included residual tumor but repeat resection yielded negative margins. If the surgical team has preoperative suspicion for liposarcoma of the spermatic cord, it may be prudent to obtain initial consent for orchiectomy. Finally, our patient’s abdominal wall defect was repaired and reinforced with biologic mesh. The choice between biologic and synthetic mesh is currently an area of debate [8]. The choice of biologic mesh was based on the exposure of the external iliac vessels during the re-resection and the assumption that biologic material may create less adhesions to these critical structures after radiation. Some authors describe the use of the ipsilateral tensor fascia lata as a tension-free flap reconstruction of the abdominal wall defect [8]. Given the high local recurrence rate of liposarcoma, possible future surgery is a consideration. Adjunct radiotherapy in dedifferentiated liposarcoma, as in our patient, is also supported by small case series and expert opinion [3,9,10]. Many tumor excisions do not achieve negative margins or have close margins due to the proximity of other structures; adjuvant radiotherapy is commonly recommended in these situations [3]. Fortunately, six months from tumor resection and adjuvant radiation, our patient is free from disease recurrence and can participate in his usual daily activities.

## 4 Conclusion

Liposarcoma of the Spermatic Cord is a rare and highly malignant tumor that rapidly recurs. Therefore, a high degree of suspicion, regular surveillance and imaging are justified.

## Declaration of Competing Interest

**Mr. Eric Wetzel** or any member of his or her immediate family, has no funding or commercial associations (e.g. consultancies, stock ownership, equity interest, patent/licensing arrangements, etc.) that might pose a conflict of interest in connection with the submitted article.

**Dr. Norair Adjamia** or any member of his or her immediate family, has no funding or commercial associations (e.g. consultancies, stock ownership, equity interest, patent/licensing arrangements, etc.) that might pose a conflict of interest in connection with the submitted article.

**Dr. Graal Diaz,** or any member of his or her immediate family, has no funding or commercial associations (e.g. consultancies, stock ownership, equity interest, patent/licensing arrangements, etc.) that might pose a conflict of interest in connection with the submitted article.

**Dr. Shawn Steen,** or any member of his or her immediate family, has no funding or commercial associations (e.g. consultancies, stock ownership, equity interest, patent/licensing arrangements, etc.) that might pose a conflict of interest in connection with the submitted article.

**Ms. Janet Hobbs,** or any member of his or her immediate family, has no funding or commercial associations (e.g. consultancies, stock ownership, equity interest, patent/licensing arrangements, etc.) that might pose a conflict of interest in connection with the submitted article.

## Funding

The authors have no source of funding to disclose.

## Ethical approval

There is no ethical approval was obtained as it’s a case report but a written consent was taken from the patient.

## Consent

Written informed consent was obtained from the patient for publication of this case report and accompanying images. A copy of the written consent is available for review by the Editor-in-Chief of this journal on request.

## Author contribution

Eric Wetzel: study concept, design, writing the paper.

Norair Adjamian: data collection, data analysis, interpretation, writing the paper.

Graal Diaz: data collection, writing the paper.

Shawn Steen: data collection, writing the paper.

Janet Hobbs: editorial review, formatting and submitting the paper

## Registration of research studies

NA.

## Guarantor

Dr. Graal Diaz.

## Provenance and peer review

Not commissioned, externally peer-reviewed.
